# Die Prävalenz an Demenz erkrankter Menschen in Deutschland – eine bundesweite Analyse auf Kreisebene

**DOI:** 10.1007/s00115-020-00923-y

**Published:** 2020-05-12

**Authors:** Jochen René Thyrian, Melanie Boekholt, Wolfgang Hoffmann, Maren Leiz, Jessica Monsees, Tim Schmachtenberg, Fanny Schumacher-Schönert, Ulrike Stentzel

**Affiliations:** 1 Standort Rostock/Greifswald, Deutsches Zentrum für Neurodegenerative Erkrankungen (DZNE), Ellernholzstr. 1–2, 17489 Greifswald, Deutschland; 2 Institut für Community Medicine (ICM), Abt.: Versorgungsepidemiologie und Community Health, Universitätsmedizin Greifswald, Greifswald, Deutschland; 3 European Network on research in dementia (INTERDEM), Nijmegen, Niederlande

## Hintergrund

Den aktuellen Zahlen zufolge leben zurzeit ca. 1,7 Mio. Menschen mit Demenz (MmD) in Deutschland, wobei jährlich mehr als 300.000 Menschen neu erkranken [1]. Diese Zahlen werden regelmäßig von der Deutschen Alzheimer Gesellschaft veröffentlicht und basieren auf Daten des Statischen Bundesamtes und den auf (europäischen) Feldstudien basierenden Prävalenzschätzungen von EuroCoDe [2]. Das bisherige Fehlen einer bundesweiten Darstellung der Ergebnisse für einzelne Regionen wird auf fehlende Evidenz zurückgeführt, da noch nicht verlässlich beurteilbar sei, ob es innerhalb eines Landes Regionen gibt, deren Bewohner unter einem besonderen Risiko stünden. Oder positiv ausgedrückt, ob deren Bewohner ein geringeres Risiko aufweisen. Evidenz aus anderen westlichen Industrieländern zeigten keine signifikanten, regionalen Schwankungen [1].

Analysen von ambulanten Abrechnungsdaten haben jedoch deutliche, regionale Unterschiede hinsichtlich der Versorgung von MmD aufgezeigt [3–5]. So wurde im Jahr 2009 in den neuen Bundesländern überdurchschnittlich oft eine Demenzdiagnose gestellt, während die Raten in Baden-Württemberg und Bayern unterdurchschnittlich ausfielen. Regionale Unterschiede zeigten sich sowohl bei der Anwendung testpsychologischer und bildgebender Verfahren (Versorgungsatlas; [5]). Bezüglich der Versorgung von MmD fällt ein Ost-West-Gefälle bei der Verschreibung von Antidementiva sowie ein West-Ost-Gefälle hinsichtlich der Verordnung von Antidepressiva und Antipsychotika auf [4]. Regional zum Teil sehr deutliche Unterschiede zeigen sich in dem Anteil der Patienten, die innerhalb von 6 Wochen nach ihrer Erstdiagnose ambulant durch Neurologen, Nervenärzte und Psychiater betreut wurden. Je mehr Neurologen, Nervenärzte und Psychiater in einem Bundesland pro 100.000 Einwohner existieren, desto mehr Demenzpatienten mit Erstmanifestation wurden innerhalb der ersten 6 Wochen fachärztlich versorgt [3].

Laut Analysen des Robert-Koch-Instituts (RKI) bestehen „zum Teil ausgeprägte regionale Ungleichheiten in der Lebenserwartung, im Auftreten von Krankheiten und gesundheitlichen Beschwerden sowie im Gesundheitsverhalten (…) Wichtige Bestimmungsfaktoren regionaler Unterschiede sind dabei insbesondere die demografische Struktur und die soziale Lage der Bevölkerung“. Das RKI weist darauf hin, dass „regionale Unterschiede in der Gesundheit mit Unterschieden hinsichtlich des Bedarfs an medizinischen Leistungen einhergehen können. Sie stellen somit Ansatzpunkte für die Prävention, den öffentlichen Gesundheitsdienst der Länder und Kommunen und die Bedarfsplanung für die ambulante, stationäre und pflegerische Versorgung dar. Das Thema ist darum von besonders hoher Public Health-Relevanz“ [6].

Für die allgemeine Planung der Versorgung von MmD auf Kreisebene sind Schätzungen hilfreich, die angeben, (a) wie viele Menschen betroffen sind, (b) wie groß deren Anteil an der Bevölkerung ist. Darüber hinaus unterscheiden sich Kreise und kreisfreie Städte hinsichtlich ihrer Bevölkerungsdichte, welches einen Einfluss auf die Versorgungsstrukturen hat [7]. So sind z. B. Anfahrtswege/-kosten oder auch die Auslastung sehr spezifischer Strukturen in ländlichen Gebieten von anderer Bedeutung als in Ballungsgebieten. Es fehlt jedoch eine Darstellung, (c) wie hoch die geografische Dichte von MmD in einzelnen Regionen in Deutschland ist.

Ziel der vorliegenden Arbeit ist eine Analyse der MmD bez. Anzahl, Bevölkerungsanteil und geografischer Dichte auf Kreis- und ein Vergleich auf nationaler Ebene.

## Methoden

Die Analysen basieren auf: a) der amtlichen Bevölkerungsstatistik des Statistischen Bundesamtes, b) den Angaben zur Prävalenz von Demenz in Deutschland basierend auf EuroCoDe ([2] zit. nach [1]), und c) der Flächenangaben des Statistischen Bundesamtes für die Kreise und kreisfreien Städte. Die amtliche Bevölkerungsstatistik beschreibt auf Ebene der *n* = 401 Kreise und kreisfreien Städte die Wohnbevölkerung zum Stichtag 31.12.2018. Diese Daten sind öffentlich verfügbar. Die Prävalenz von Demenz in Deutschland wurde analog den EuroCoDe-Daten geschätzt; verwendet wurden die altersgruppen- und geschlechtsspezifischen Schätzungen der Menschen ab 65 Jahren (Tab. 1). Aufgrund fehlender, zuverlässiger Daten zur Prävalenz an Demenz erkrankter jüngerer Menschen wird diese Personengruppe in dieser Analyse nicht berücksichtigt. Die Information über die geografische Größe der Kreise und kreisfreien Städte in km^2^ ist ebenfalls öffentlich verfügbar und kann über das Statistische Bundesamt bezogen werden.

**Table 1:** 

Altersgruppe	Gesamt	Männer	Frauen
*65–69*	1,6	1,79	1,43
*70–74*	3,50	3,23	3,74
*75–79*	7,31	6,89	7,63
*80–84*	15,60	14,35	16,39
*85–89*	26,11	20,85	28,35
*90 und älter*	40,95	29,18	44,17
*65 und älter*	9,99	7,16	10,95

Berechnet wurde für jede geografische Einheit: 1. Die *Anzahl* der an Demenz erkrankten Personen – dies geschah durch Multiplikation der Einwohner und der angenommenen Prävalenz der Demenz in den einzelnen Altersgruppen; 2. *Bevölkerungsanteil*: der prozentuale Anteil an Demenz erkrankter Menschen an der Gesamtbevölkerung; 3. Die Anzahl der Menschen mit Demenz pro Fläche in Quadratkilometer.

Die Darstellung der Ergebnisse erfolgt in Tabellenform, wie auch als geografische Karte, die mithilfe der Software ArcGIS (ESRI®ArcGIS™ 10.0 Esri Inc., Redlands/California, USA) entworfen wurde. Für diese geografische Darstellung wurden spezifische Kategorien gebildet, die in den Grafiken angegeben werden. Die Kategorien sind zum einen mit ungefähr gleich vielen Kreisen besetzt, zum anderen aber an runden Zahlen orientiert. Eine Kategorisierung mit gleichen Abständen wäre zum einen zu unübersichtlich aufgrund einer hohen Anzahl an Kategorien, zum anderen weniger aufschlussreich aufgrund des Informationsverlustes durch eine geringe Anzahl an Kategorien.

## Ergebnisse

**Figure 1:**
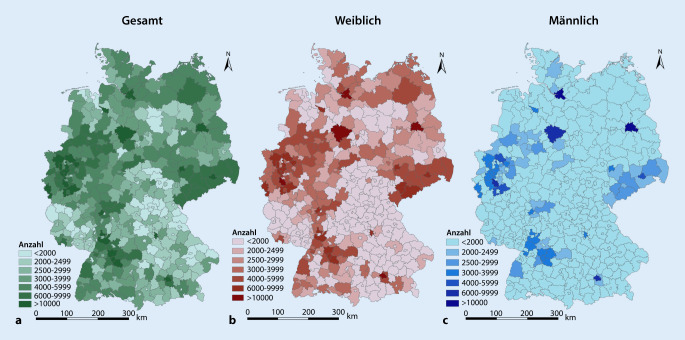

Insgesamt wird die Anzahl der MmD in Deutschland zum Stichtag 31.12.2018 auf 1.699.785 geschätzt. Auf Kreisebene variiert sie von *n* = 780 (kreisfreie Stadt Zweibrücken) bis *n* = 64.188 (kreisfreie Stadt Berlin). Das arithmetische Mittel beträgt *n* = 4239, der Median *n* = 3115. Detaillierte Angaben zu allen Kreisen und kreisfreien Städten finden sich in Tab. 2 im Zusatzmaterial online. Eine Betrachtung der kartografischen Darstellung in Abb. 1 zeigt wie zu erwarten hohe Anzahlen in den Städten und Ballungsgebieten in Deutschland.

**Figure 2:**
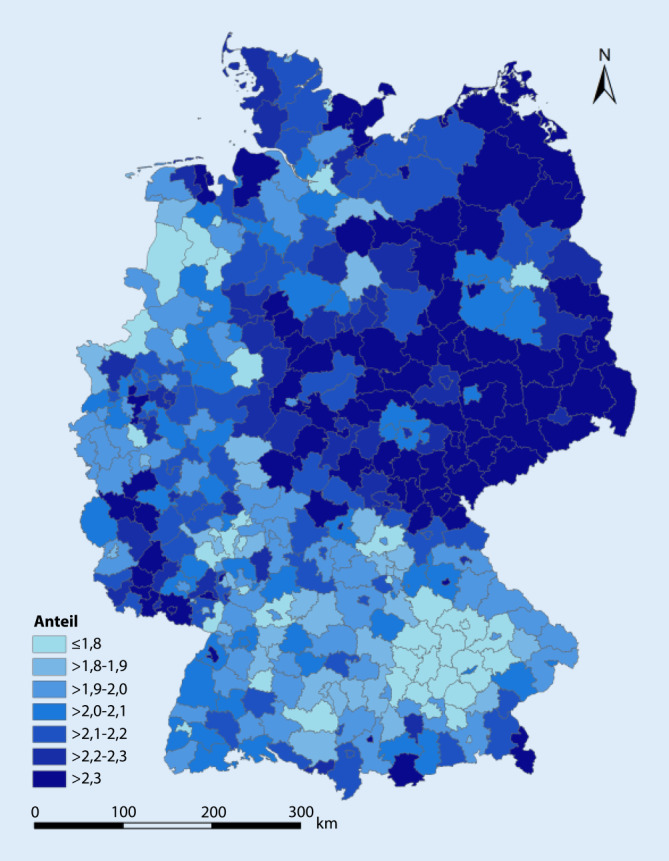

Der Anteil der MmD an der Gesamtbevölkerung in Deutschland beträgt 2,04 %. Auf Kreisebene variiert er zwischen 1,42 % (Landkreis Freising) und 3,01 % (kreisfreie Stadt Dessau-Roßlau). Das arithmetische Mittel beträgt 2,12 %, der Median 2,09 %. Die kartografische Darstellung in Abb. 2 zeigt einen höheren Anteil an der Gesamtbevölkerung der Kreise in einigen östlichen Bundesländern sowie im Norden und im mittleren Südwesten. Detaillierte Angaben zu allen Kreisen und kreisfreien Städten finden sich in Tab. 2 im Zusatzmaterial online.

**Figure 3:**
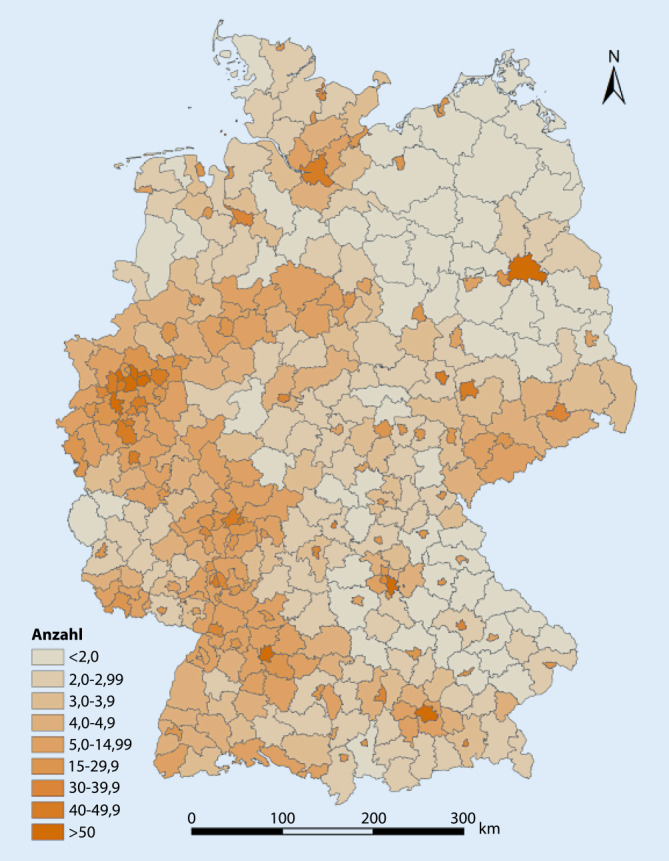

Die geschätzte Anzahl von MmD pro Quadratkilometer in Deutschland beträgt 4,75. Auf Kreisebene variiert sie zwischen 0,8 (Altmarkkreis Salzwedel) und 78,5 (München). Das arithmetische Mittel beträgt 10,8, der Median 4,2. Die kartografische Darstellung in Abb. 3 zeigt die entsprechenden Unterschiede für Deutschland. Detaillierte Angaben zu allen Kreisen und kreisfreien Städten finden sich in Tab. 2 im Zusatzmaterial online.

## Diskussion

Die Versorgung von MmD in Deutschland muss vor dem Hintergrund regionaler Unterschiede gestaltet und geplant werden. So zeigen unsere Analysen, dass eine Darstellung auf Bundesebene die Gegebenheiten für die einzelnen Kreise und kreisfreien Städte nur unzureichend abbildet. Während z. B. in den Kreisen Freising und Frankfurt/Main der Anteil an Demenz erkrankter Menschen an der Bevölkerung <1,6 % liegt, so ist dieser Anteil in Görlitz oder Dessau-Roßlau >2,9 % (teilweise doppelt so hoch). Auch bei der geschätzten Anzahl von MmD in den einzelnen Kreisen wird sichtbar, welchen Stellenwert die Versorgung von MmD haben sollte. So liegen die Schätzungen in kleineren Kreisstädten wie z. B. Zweibrücken, Schwabach, Landau i.d. Pfalz oder Ansbach weit unter 1000 MmD, während diese für 23 Kreise (meist Städte) über 10.000 MmD hinausgehen.

Darüber hinaus denken wir, dass die Maßzahl von MmD pro km^2^ ein differenzierendes Merkmal bei der Versorgungsplanung sein könnte. Es ist davon auszugehen, dass in ländlichen Kreisen wie z. B. Salzwedel oder Ostprignitz-Ruppin bei weniger als einem MmD pro km^2^ die wohnortnahe Versorgung schwieriger, zumindest aber anders aussehen muss als in Kreisen wie Oldenburg, Flensburg, M’gladbach oder Chemnitz, die eine 30-fach höhere Rate aufweisen. Es scheint offensichtlich, dass z. B. spezialisierte, ambulante Angebote eine höhere Wahrscheinlichkeit der Inanspruchnahme haben, je mehr MmD im Einzugsgebiet wohnen. Auch vor dem Hintergrund der aufgrund des Alters eingeschränkten Mobilität der älteren und betagten Bevölkerung ist die Frage zu beantworten, inwieweit vergleichbare Lebensbedingungen geschaffen werden können, die keine Abstriche bei einer qualitativ hochwertigen, wohnortnahen, spezialisierten Versorgung aufweisen. Weitere Analysen müssen zeigen, welche Art der Versorgung für welche Regionen passt.

## Limitationen

Unsere Analysen basieren auf der zurzeit verlässlichsten Datengrundlage; diese sind allerdings Schätzungen deren Aussagekraft Einschränkungen unterliegt.

Kreise sind keine homogenen, distinkten Regionen. Auch innerhalb von Regionen gibt es Ballungsgebiete oder eher ländliche Gebiete bzw. Viertel mit unterschiedlichen Altersstrukturen. Ebenso grenzen manche Landkreise an kreisfreie Städte. Versorgung findet über die kommunalen Grenzen hinweg statt, gerade spezialisierte Einrichtungen in Ballungsgebieten haben Einzugsgebiete, die weit über diese Grenzen hinausgehen (Edge-Effekt). Insofern sind Aussagen auf der hier betrachteten kommunalen Ebene ohne die Kenntnis der örtlichen Gegebenheiten vorsichtig zu treffen.

Die Ergebnisse hängen stark mit der Altersverteilung und der Bevölkerungsdichte in den einzelnen Kreisen und kreisfreien Städten zusammen. Aus diesem Grund sind die einzelnen Befunde dieser Analyse nicht überraschend. Es existiert jedoch zum ersten Mal für Gesamtdeutschland eine umfassende Darstellung der erhobenen Parameter auf Kreisebene. Dies kann Grundlage für weitere Analysen sein, wie z. B. Prognosen zu Entwicklungen auf Kreisebene und zur Verbesserung der kommunalen Bedarfsplanung.

## Caption Electronic Supplementary Material


